# Heterogeneity Matters: Aggregation Bias of Gas Transfer Velocity Versus Energy Dissipation Rate Relations in Streams

**DOI:** 10.1029/2021GL094272

**Published:** 2021-09-08

**Authors:** Gianluca Botter, Paolo Peruzzo, Nicola Durighetto

**Affiliations:** ^1^ Department of Civil, Environmental, and Architectural Engineering University of Padua Padova Italy

**Keywords:** aggregation bias, gas transfer velocity, energy dissipation rate, gas exchange, reaeration, scaling

## Abstract

The gas transfer velocity, k, modulates gas fluxes across air‐water interfaces in rivers. While the theory postulates a local scaling law between k and the turbulent kinetic energy dissipation rate ε, empirical studies usually interpret this relation at the reach‐scale. Here, we investigate how local k(ε) laws can be integrated along heterogeneous reaches exploiting a simple hydrodynamic model, which links stage and velocity to the local slope. The model is used to quantify the relative difference between the gas transfer velocity of a heterogeneous stream and that of an equivalent homogeneous system. We show that this aggregation bias depends on the exponent of the local scaling law, b, and internal slope variations. In high‐energy streams, where b>1, spatial heterogeneity of ε significantly enhances reach‐scale values of k as compared to homogeneous settings. We conclude that small‐scale hydro‐morphological traits bear a profound impact on gas evasion from inland waters.

## Key Points

- We analyze local and reach‐wise relations between gas transfer velocity (*k*) and energy dissipation rate (*ε*)
- Reach‐scale *k*(*ε*) laws depend on the exponent that modulates the local relation between *k* and *ε* and the heterogeneity of the reach
- Reach‐scale *k*(*ε*) laws in high‐energy heterogeneous streams are affected by a positive aggregation bias

## 1 Introduction

Freshwater systems are a key component of the global carbon and nitrogen cycle (Battin et al., 2009; Bernhardt et al., 2018; Galloway et al., 2004; Hotchkiss et al., 2015; Marx et al., 2017; Raymond et al., 2013). In particular, headwater streams not only represent an important source of nutrients for downstream ecosystems but—owing to the high energy of the flow—they are also responsible for significant green‐house gas (e.g., $CO_{2}$, $CH_{4}$, and $N_{2}O$) emissions to the atmosphere (Crawford et al., 2014; Kroeze et al., 2005; Hall & Ulseth, 2020; Horgby et al., 2019; Marzadri et al., 2017; Schelker et al., 2016). Thus, an accurate quantification of water‐air gas exchanges in low‐order streams proves crucial to better constrain current estimates of nutrient loads to recipient water bodies and river outgassing at regional or global scales (Rawitch et al., 2019).

One of the key parameters that control gas evasion in streams and rivers is the transfer velocity k, which represents the water depth that equilibrates with the atmosphere per unit time. k regulates the relation between gas fluxes across the air‐water interface and the concentration gradient across the water boundary layer, as per the Fick's first law of diffusion (Vingiani et al., 2021; Wanninkhof et al., 2009). The gas transfer velocity k is a local property of the flow field, which reflects the intensity of the near‐surface turbulence and renewal rates in the water column (Zappa et al., 2003). Periodically, in fact, the fluid from the water column replaces the liquid film on the free surface where the exchange of gas with the atmosphere occurs. In streams and rivers, this circulation is typically related to the small eddies generated by the local turbulence at the Batchelor‐scale, in which k is proportional to $Sc{\left(\varepsilon \;\nu \right)}^{0.25}$, where Sc=ν/D is the Schmidt number, ν is the kinematic fluid viscosity, D is the molecular diffusivity of the dissolved gas, and ε is the energy dissipation rate per unit mass. In the above formulation, the Batchelor length scale, and thus gas evasion, depend on turbulence characteristics, gas diffusivity and temperature via ε and Sc. The power‐law scaling of k with ε is well established in the literature, and the theoretical value of the scaling exponent (0.25) is supported by many in‐field and laboratory experiments (Katul & Liu, 2017; Lamont & Scott, 1970; Moog & Jirka, 1999a). However, recent works (Ulseth et al., 2019) have proposed the existence of a super‐linear scaling between k and ε in high‐energy streams, which arguably reflects bubble‐mediated processes promoted by air entrainment (Chanson et al., 2000).

Most of the available experimental methods to measure k in the field—such as tracer experiments, night‐time regression of dissolved oxygen data, coupled estimation of daily metabolic rates—provide information about reach‐scale values of the gas transfer velocity. Consequently, available estimates of k subsume spatial scales ranging from several tens to hundreds of meters—with shorter lengths that are typically associated with high‐energy streams, where gas exchange is enhanced (Bernot et al., 2010; Bott et al., 2006; Melching & Flores, 1999; Mulholland et al., 2001; Tsivoglou & Neal, 1976; Ulseth et al., 2019). While local estimates of k are also feasible (e.g., via steady floating chambers, see Vingiani et al., 2021), reach‐scale estimates are particularly important for practical purposes, as long as they provide an objective basis for the upscaling of mass transfer rates at the network level (Bertuzzo et al., 2017; Raymond et al., 2013).

Owing to the spatial scales involved, experimental k measurements usually refer to reaches that are featured by a strong internal heterogeneity. This lack of homogeneity of the flow field is particularly pronounced in high‐energy streams, which are often characterized by complex and diversified morphologies even at relatively short spatial scales (e.g., step and pools, slope changes, presence of hurdles within the flow, small cascades, dead‐end zones). Accordingly, experimental scaling laws between the mass transfer velocity and the energy dissipation rate available in the existing literature involve reach‐wise values of k and ε—the latter being calculated from mean characteristics of the flow field (Moog & Jirka, 1999b; Raymond et al., 2012). Although empirical k versus ε relations necessarily disregard the internal complexity of streams and rivers, the mechanistic link between the energy dissipation processes and the gas transfer velocity has an inherently local nature, and observed patterns of gas evasion within composite reaches properly reflect local heterogeneity of in‐stream hydrodynamic conditions (Kokic et al., 2018; Leibowitz et al., 2017; Vautier et al., 2020).

As the link between k and ε is nonlinear (i.e., $k\propto \varepsilon^{b}$ with b≠1), spatial integrations along heterogeneous domains can alter significantly the nature of their relation (i.e., $\langle \varepsilon^{b}\rangle \neq {\langle \varepsilon \rangle }^{b}$), thereby making empirically derived k(ε) laws potentially scale‐dependent. Accordingly, the main research hypothesis of the present work is that reach‐scale gas transfer versus total kinetic energy dissipation rates relations bear the signature of the spatial heterogeneity of the flow filed typical of many real‐world settings. This internal heterogeneity of river systems lies at the basis of a systematic aggregation bias affecting observed reach‐scale k versus ε laws. This study combines a simple hydrodynamic model and a novel probabilistic framework to quantify and correct such bias using measurable external variables such as slope variations.

## 2 Theory

### 2.1 Hydrodynamic Setup and Local Turbulent Kinetic Energy Dissipation Rate

**Figure 1:**
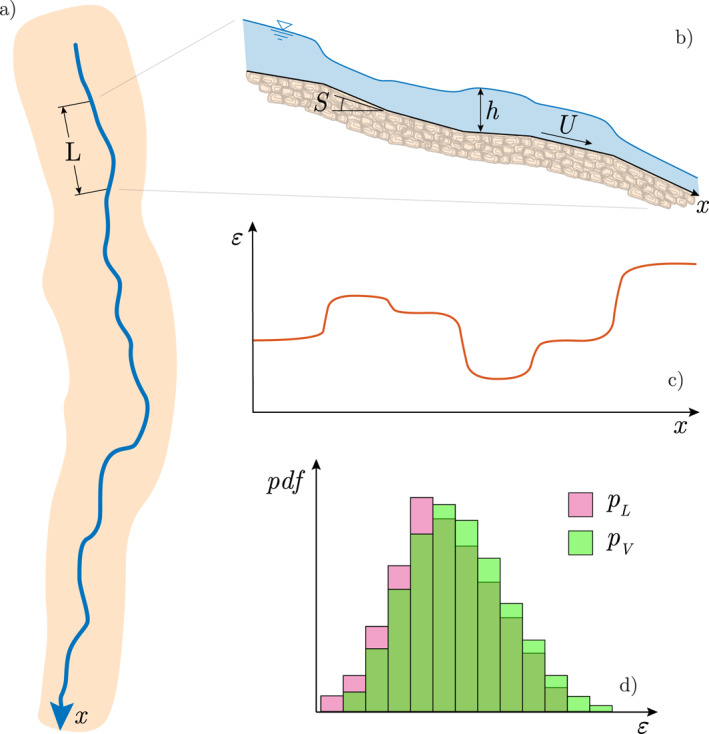
Schematic representation of a stream reach: map view (a), longitudinal profile of the elevation (b), and spatial patterns of energy dissipation rate, ε (Equation 3) (c). Panel (d) shows the probability density functions of ε within the reach (Equations 6 and 7).

We focus on the one‐dimensional (1D) modeling of a rectangular cross‐sectional stream with a total length L, here representing the length of an heterogeneous stream within which spatially integrated estimates of k are available (e.g., tens to hundreds of meters—depending on the specific case‐study). Owing to the assumptions formulated hereafter, the minimum value of L is ideally set to some tens of meters—although the general idea behind this study could be extended also to shorter reach segments. Regardless of the specific value of L, we assume that owing to internal variations in the slope the turbulent kinetic energy dissipation rate ε (i.e., the power dissipated per unit mass by the flow) and the gas transfer velocity k are continuous functions of x—here representing the position along the considered stream path (Figure 1). To simplify the problem description, the impact of heat fluxes and temperature variations along the stream is here neglected, that is, Sc≃const(x). In addition, we also assume that the cross‐section width (W), the discharge (Q) and the bed roughness are constant along the focus reach. The hydraulic regime is then conceptualized as a sequence of piecewise nearly uniform flows, in which the possible transitions from supercritical to subcritical flow conditions (or vice versa) are neglected. In this setting, the changes in the bed slope S(x) induce local changes in the cross‐sectional velocity U(x), according to the following relation:

(1) $$U=\xi \cdot {R_{H}}^{\eta }\cdot S^{1/2}\approx \xi \cdot h^{\eta }\cdot S^{1/2},$$

where η is a positive exponent, h is the water dept, and ξ is a coefficient strictly related to the bed friction. In Equation 1, $R_{H}=A/\omega$ is the hydraulic radius, that is, the ratio between the cross‐sectional area A=Wh and the wetted perimeter ω=W+2h. For h/W≪1, U simplifies as indicated in the r.h.s of Equation 1 because $R_{H}\approx h$. The nature of the above equation sets the spatial resolution of the model to a minimum value of a few meters. As the discharge Q can be expressed as Q=WU(x)h(x), we can rearrange Equation 1 and express the water depth, h, as a function of the slope as:

(2) $$h={\left(\frac{Q}{\xi \;W}\right)}^{2d}\;S^{-d},$$

where $d={\left(\eta +1\right)}^{-1}/2$ is a coefficient strictly related to the particular empirical model chosen for Equation 1. For instance, if we specify Equation 1 according to the Manning's formula, we have ξ=1/n (where n is the Manning's coefficient) and η=2/3, thereby leading to d=0.3.

According to Equations 1 and 2, both U and h vary with S. This originates spatial variations in the energetic configuration of the flow, which are quantified by the turbulent kinetic energy dissipation rate of the bulk flow, ε(x). Under the assumption of uniform flow, ε(x) can be approximated as (Tsivoglou & Neal, 1976):

(3) ε(x)=gS(x)U(x),

where g is the gravitational acceleration. A word of caution is needed at this point. The proposed 1D formulation neglects the vertical profile of turbulent kinetic energy dissipation rate within each cross section (Moog & Jirka, 1999b), and implicitly assumes that the value of ε driving the gas exchange at the water‐air interface can be approximated by the average value of ε in the bulk flow, as given by Equation 3. This choice is in line with the large majority of the experimental datasets currently available for streams and rivers (e.g., Raymond et al., 2012; Ulseth et al., 2019), and is suited to describe cases in which the gas exchange takes place over the entire water column, for example, owing to bubble‐mediated exchange and air entrainment.

Combining Equations (1), (2), (3), the following expression for ε is obtained:

(4) $$\varepsilon \left(S\right)=\varepsilon_{1}\;S^{1+d},$$

where $\varepsilon_{1}=g\;{\left(Q/W\right)}^{1-2d}\;\xi^{2d}$ is a constant, corresponding to the energy dissipation rate for a unit slope.

### 2.2 Characterizing Spatial Variations of Total Kinetic Energy Dissipation Rates

The spatial variability of ε is here described through its probability density function (pdf), which quantifies how often different values of ε are observed within the focus reach. In this framework, two different though related pdfs need to be introduced to quantify the spatial patterns of ε (Figure 1d):

1. The longitudinal pdf of ε, $p_{L}\left(\varepsilon \right)$, which is the longitudinal pdf of the energy dissipation rate ε along the stream. This is simply the pdf of the values of ε observed within the different cross sections of the focus river reach. $p_{L}\left(\varepsilon^{*}\right)d\varepsilon^{*}$ is the probability that ε(x)(x∈(0,L)) belongs to the interval ($\varepsilon^{*},\;\varepsilon^{*}+d\varepsilon^{*}$)—which also corresponds to the fraction of length of the reach for which $\varepsilon \in \left(\varepsilon^{*},\;\varepsilon^{*}+d\varepsilon^{*}\right)$; and
2. The volumetric pdf of ε, $p_{V}\left(\varepsilon \right)=p_{L}\left(\varepsilon \right)h\left(\varepsilon \right)/\langle h\rangle$ where h(ε) is the bijective function that relates the water stage and the energy dissipation rate (see Equations 2 and 4) and 〈h〉 is the mean stage along the focus reach. This is the volumetric pdf of the energy dissipation rate ε within the reach, and differently from $p_{L}\left(\varepsilon \right)$ attributes a larger weight to the values of ε that pertain to the regions of the domain where the stage is higher. Accordingly, $p_{V}\left(\varepsilon^{*}\right)d\varepsilon^{*}$ is the fraction of water volume contained in the reach with a value of ε in the interval ($\varepsilon^{*},\;\varepsilon^{*}+d\varepsilon^{*}$).

Owing to the link between ε and S (Equation 4), the two pdfs of ε introduced above can be calculated starting from the longitudinal pdf of the slope along the focus reach, $p_{S}\left(S\right)$. In line with observational evidence and previous studies, S was here assumed to follow a two‐parameters Gamma distribution (Tarboton et al., 1989):

(5) $$p_{S}\left(S\right)=\frac{\alpha^{-\beta }}{\Gamma \left(\beta \right)}\;S^{\beta -1}\;exp\left(-\frac{S}{\alpha }\right),$$

where the shape parameter α and the scale parameter β constrain the moments of S. In particular, β is the inverse of the square of the coefficient of variation of the slope, that is, $\beta ={\left[CV_{S}\right]}^{-2}$. The longitudinal pdf of the kinetic energy dissipation rates, $p_{L}\left(\varepsilon \right)$ can be obtained from Equations 4 and 5 as:

(6) $$p_{L}\left(\varepsilon \right)=\;\frac{\;\alpha^{-\beta }}{\varepsilon_{1}\;\left(1+d\right)\;\Gamma \left(\beta \right)}\;{\left(\frac{\varepsilon }{\varepsilon_{1}}\right)}^{\frac{\beta }{1+d}-1}\;exp\left(-\frac{1}{\alpha }\;{\left(\frac{\varepsilon }{\varepsilon_{1}}\right)}^{\frac{1}{1+d}}\right).$$

Likewise, the volumetric pdf of ε can be obtained combining the relation between $p_{V}$ and $p_{L}$ and Equation 6 as:

(7) $$p_{V}\left(\varepsilon \right)=\frac{\;\alpha^{d-\beta }}{(1+d)\;\varepsilon_{1}\;\Gamma \left(\beta -d\right)}\;{\left(\frac{\varepsilon }{\varepsilon_{1}}\right)}^{\frac{\beta -d}{d+1}-1}\;exp\left(-\frac{1}{\alpha }\;{\left(\frac{\varepsilon }{\varepsilon_{1}}\right)}^{\frac{1}{1+d}}\right).$$

These pdfs express the probability distribution functions of the total kinetic energy dissipation rate within the focus reach, as a function of four parameters: two morphological parameters that define the internal heterogeneity of the slope (α and β), and two hydrodynamic parameters ($\varepsilon_{1}$ and d).

### 2.3 Upscaling Total Kinetic Energy Dissipation Rates

Let us consider the 1D stream reach of length L depicted in Figure 1. The total power dissipated within the reach, P, can be expressed by integrating along the reach the product between the dissipation rate ε(x), and the related water mass per unit length, that is,:

(8) $$P=\int_{0}^{L}\rho A\left(x\right)\varepsilon \left(x\right)dx,$$

where ρ is the water density. The spatial patterns in the local energy dissipation rate ε(x) originate a reach‐wise equivalent counterpart, which is here denoted as $\varepsilon_{eq}$. The equivalent energy dissipation rate, $\varepsilon_{eq}$, should provide the same total energy losses induced by the assortment of local ε(x) experienced by the flow along the reach. The total dissipated power can be written in terms of $\varepsilon_{eq}$ as:

(9) $$P=\rho \langle A\rangle L\varepsilon_{eq},$$

where 〈A〉 is the average cross sectional area of the stream reach. By equaling Equations 8 and 9, the equivalent energy dissipation rate can be written as:

(10) $$\varepsilon_{eq}=\frac{1}{\langle A\rangle L}\int_{0}^{L}\varepsilon \left(x\right)A\left(x\right)dx=\int_{0}^{\infty }\varepsilon \;p_{V}\left(\varepsilon \right)d\varepsilon =\langle {\varepsilon \rangle }_{V},$$

which corresponds to a weighted average of ε(x) over the stream length, the weights being set by the volumetric pdf of ε in the considered reach, $p_{V}\left(\varepsilon \right)$.

A more explicit formulation for $\varepsilon_{eq}$ can be obtained by inserting Equations 3 into 10 and recalling that U(x)=Q/A(x), that is,:

(11) $$\langle {\varepsilon \rangle }_{V}=\frac{g\;Q}{\langle A\rangle L}\int_{0}^{L}\;S\left(x\right)dx=g\frac{Q}{\langle A\rangle }\langle S\rangle ,$$

where $Q{\langle A\rangle }^{-1}$ is the mean water velocity measured by means of tracer injections.

### 2.4 Upscaling Gas Transfer Velocity

The concentration of a conservative gas in water is governed by the equation:

(12) $$\frac{\partial C\left(x,t\right)}{\partial t}=-U\left(x\right)\frac{\partial C\left(x,t\right)}{\partial x}+\frac{\partial }{\partial x}\left[D_{x}\left(x\right)\frac{C\left(x,t\right)}{\partial x}\right]-\frac{k\left(x\right)}{h\left(x\right)}\left(C\left(x,t\right)-C_{a}\right),$$

where $D_{x}$ is the hydrodynamic dispersion, C is the gas concentration in water, $C_{a}$ is the corresponding equilibrium concentration (the concentration that equilibrate with the atmosphere) and k(x) is the gas transfer velocity. If the dispersion term is neglected (i.e., $D_{x}\approx 0$), as typically done in experimental tracer studies (e.g., Benson et al., 2014; Heilweil et al., 2016; Ulseth et al., 2019), the general solution of Equation 12 reads:

(13) $$C\left(x,t\right)=C_{a}+\left[C_{0}\left(t-\int_{0}^{x}1/U\left(x^{\prime }\right)dx^{\prime }\right)-C_{a}\right]exp\left(-\frac{W}{Q}\int_{0}^{x}k\left(x^{\prime }\right)dx^{\prime }\right),$$

where $C_{0}$ is the time‐dependent gas concentration in x=0. Equation 13 shows how spatial variations of the local gas transfer velocity k(x) determine the patterns of downstream gas concentration. During field experiments, the internal heterogeneity of the studied stream is usually neglected and an equivalent reach‐scale gas transfer rate $k_{eq}$ is estimated.

The relation between $k_{eq}$ and k(x) can be found by equaling the concentration in the downstream section of the reach, C(L,t), under the following conditions: (a) the case of an heterogeneous gas transfer velocity, k(x), which corresponds to Equation 13 calculated for x=L; and (b) the case of a spatially uniform gas transfer velocity, which corresponds to Equation 13 calculated for x=L with $k\left(x\right)=k_{eq}=const$. This equality leads to the following expression for $k_{eq}$:

(14) $$k_{eq}=\frac{1}{L}\int_{0}^{L}k\left(x\right)dx={\langle k\rangle }_{L}.$$

Equation 14 shows that the equivalent mass transfer velocity is a spatial longitudinal average of the local gas transfer velocities along the stream.

### 2.5 Upscaling Gas Transfer Velocity Versus Total Kinetic Energy Dissipation Rate Relations

Theoretical analysis and empirical results have long suggested the existence of a power‐law relation between k and ε, of the type $k\left(\varepsilon \right)=a\;\varepsilon^{b}$. While theoretical arguments suggest that b=0.25, there is no consensus on the value of the scaling exponent and empirical data suggest that bubble‐mediated transport process could significantly increase b up to 1.2 (Ulseth et al., 2019). Although these data mostly refer to reach‐scale estimates, they likely reflect the fact that the local scaling exponent might be higher than the theoretical value of 0.25 in case of air entrainment. For these reasons, here we treat b as a model parameter. Regardless of the specific value of the scaling exponent, the k(ε) relation has an inherent local nature implied by the mechanistic link among energy dissipation, turbulence, and gas exchange at the interface.

Nevertheless, such scaling laws have been empirically analyzed at the scale of entire reaches, including highly heterogeneous river segments. Using Equation 14, the equivalent mass transfer rate for a reach with length L, $k_{eq}$, can be written as:

(15) $$k_{eq}={\langle k\rangle }_{L}=\int_{0}^{\infty }k\left(\varepsilon \right)\;p_{L}\left(\varepsilon \right)d\varepsilon =a\int_{0}^{\infty }\varepsilon^{b}\;p_{L}\left(\varepsilon \right)d\varepsilon \;=a{\langle \varepsilon^{b}\rangle }_{L}.$$

In Equation 15, the coefficient a—which depends on the features of the underlying eddy dynamics—was assumed to be spatially uniform as we expect the turbulent structures to be weakly heterogeneous along the path. Equation 15 quantifies the equivalent gas transfer rate of an heterogeneous stream with a mean value of the total kinetic energy dissipation rate equal to ${\langle \varepsilon \rangle }_{V}$. This equivalent mass transfer rate, $k_{eq}$, is equal to the $b^{th}$ order moment of ε, seen as spatial random variable with a longitudinal pdf $p_{L}\left(\varepsilon \right)$. Were the local‐scale relation between k and ε preserved at the reach level, the r.h.s. of Equation 15 should read $a\;{\langle \varepsilon \rangle }_{V}^{b}$. As in the general case ${\langle \varepsilon^{b}\rangle }_{L}\neq {\langle \varepsilon \rangle }_{V}^{b}$, Equation 15 shows that the local scaling law between k and ε is in fact altered when it is aggregated and re‐interpreted at the reach level, unless the pdf of ε is a Dirac delta distribution (i.e., the slope is spatially uniform within the reach). The resulting aggregation bias, e is quantified as the relative difference between the reach scale gas transfer velocity of an internally heterogeneous stream (Equation 15) and the gas transfer velocity of an equivalent homogeneous stream with the same mean turbulent kinetic energy dissipation rate ($a\;{\langle \varepsilon \rangle }_{V}^{b}$). Under the assumptions made, the following analytical expression for e can be obtained (see Supporting Information):

(16) $$e=\frac{a{\langle \varepsilon^{b}\rangle }_{L}-a{\langle \varepsilon \rangle }_{V}^{b}}{a{\langle \varepsilon \rangle }_{V}^{b}}=\frac{\Gamma \left(\beta +b+bd\right)}{\Gamma \left(\beta \right)}\;{\left(\frac{\Gamma \left(\beta -d\right)}{\Gamma \left(\beta +1\right)}\right)}^{b}-1.$$

Interestingly, Equation 16 shows that the bias does not depend on the discharge, Q, and the friction coefficient, ξ, but is only a function of the following independent parameters: (a) the exponent of the local scaling law that links the mass transfer rate to the total kinetic energy dissipation rate, b; (b) the hydraulic exponent d, which quantifies the relation between the hydraulic radius and slope; and (c) the parameter β, which quantifies the relative variations of the slope within the focus reach ($\beta =CV_{S}^{-2}$).

**Figure 2:**
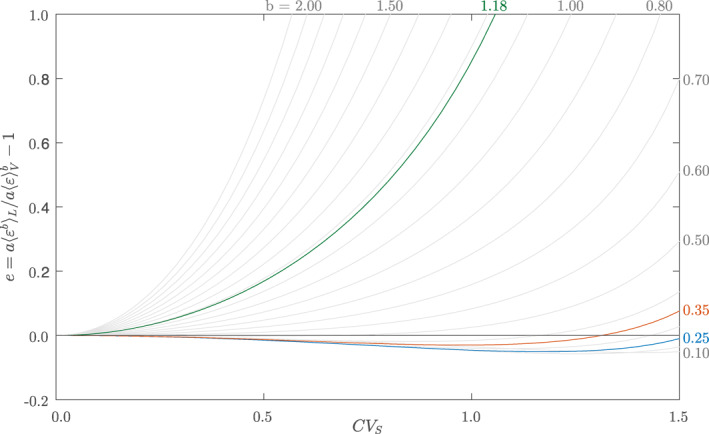
Estimation bias as a function of the coefficient of variation $CV_{S}$ and the exponent of the local scaling law, b. The blue line refers to the theoretical scaling exponent of 0.25, the orange line refers to the observed scaling exponent in low energy streams as given by Ulseth et al. (2019) (0.35) and the green line refers to the observed scaling exponent in high‐energy streams as given by Ulseth et al. (2019) (1.18).

In Figure 2, we have represented the aggregation bias e as a function of the coefficient of variation of the slope and the exponent b of the local scaling law k(ε) in the case d=0.3. Similar plots can be obtained for different values of d. The plot shows that for b>0.5 the bias is positive, and increases nonlinearly for more pronounced internal variations of the bed slope. When the variability of the slope is limited ($CV_{S}<0.5$), e is significant only when b exceeds 1. When the internal variations of the slope are moderate ($0.5<CV_{S}<1$), instead, the aggregation bias is important only for b>0.7. For values of b in the range (0.5−0.6), |e| exceeds 10% only when $CV_{S}$ is above 1, whereas for b<0.5 the magnitude of the bias is usually negligible. Interestingly, for b=1/4 (which is the expected scaling exponent according to the theory), the bias is negative up to $CV_{S}=1.5$. The behavior shown in Figure 2 is generated by the combined action of two independent components that are responsible for generating the overall aggregation bias. The first component is termed *volumetric bias component* and provides a positive contribution to the bias due to the difference between longitudinal and volumetric averages of ε in a stream, with ${\langle \varepsilon \rangle }_{L}>{\langle \varepsilon \rangle }_{V}$ in nonhomogeneous reaches; the second component can be termed *nonlinearity bias component* and it relates to the difference between the $b^{th}$ order moment of a random variable and the $b^{th}$ power of its mean, with the latter being smaller (larger) than the former for b<1 (b>1). Thus, a negative bias is observed only when b≪1 and the bias is dominated by the nonlinearity component, which is negative in this case. In all the other instances, instead, the bias is positive, as shown by Figure 2.

Overall, the analysis suggests that the aggregation bias can be neglected in low energy streams where the scaling exponent is small (theoretical value of b=0.25, observed value of b=0.35, see Zappa et al., 2007 and Ulseth et al., 2019). In those settings, the aggregation bias might be particularly low also because of the limited slope variations, which characterize most of the low‐energy streams where tracer gas experiments were performed, especially in cases when the analyzed reaches are relatively short. The limited effect of the aggregation bias for these systems is explained by the compensating effects associated with the two constitutive components of the bias. Instead, the aggregation bias is particularly important within high‐energy streams, where the scaling exponent b is higher and the coefficient of variation of the slope can be of the order of 1. Therefore, a specific real‐world example pertaining to this class of streams will be further discussed with the aid of numerical simulations in the next section.

## 3 Numerical Simulations

A real‐world headwater catchment in the Italian Alps was analyzed to quantify the spatial patterns of S(x), U(x), h(x), ε(x) and the corresponding aggregation bias in a representative high‐energy stream. The selected case study is the Rio Valfredda, which drains a 5.3 km^2^ catchment in the Alps of northern Italy. The elevation ranges from 1,500 to 3,000 m a.s.l., generating significant spatial heterogeneity in lithology and soil cover. The alpine climate typical of the area is characterized by short rainy summers and long rigid winters with significant snowfall. The average annual precipitation is 1,500 mm unevenly distributed along the year, with low flows in winter and higher discharges during spring and summer. The stream network is 16 km long and reaches the fourth Strahler order. The channel slope averages at 26.7%, with maximum values of 125% in the northern and eastern parts of the network. The river bed composition is made up of rock emergencies, gravel, and silt, while the channel width ranges from 30 cm to 1.5 m. A LiDAR survey was carried out in 2018 to generate a Digital Terrain Model (DTM) with a 20 cm resolution. For more information about the catchment, the reader is referred to Durighetto et al. (2020) and Botter and Durighetto (2020).

The aggregation bias in the Valfredda creek was estimated by means of a numerical application of the hydrodynamic model within 200 reaches with a fixed length, L, of 100 m and a random position along the network. The value of L was chosen to represent the typical length of the reaches where tracer studies are performed in high‐energy settings, though alternative choices would have led to analogous results. For each reach, the longitudinal profile was reconstructed from the DTM, and the spatial patterns of local slope were calculated with a resolution of 2 m. Considering the bed composition of the Rio Valfredda (and the related roughness), the uniform flow is assumed to be supercritical within most of the analyzed stream network (Froude number is >1 within 95% of the network length). Equations 2 and 4 were used to calculate h(x) and ε(x) along each river reach, assuming d=0.3. The local gas transfer rate k(x) was then calculated using the law proposed by Ulseth et al. (2019) for high‐energy streams: $k=6.43\;\varepsilon^{\;1.18}$. Equations 10 and 14 were employed to calculate the reach‐wise values of ${\langle \varepsilon \rangle }_{V}$ and ${\langle k\rangle }_{L}$. Finally, the aggregation bias was calculated as $e={\langle k\rangle }_{L}/\left(6.43{\langle \varepsilon \rangle }_{V}^{1.18}\right)-1$.

**Figure 3:**
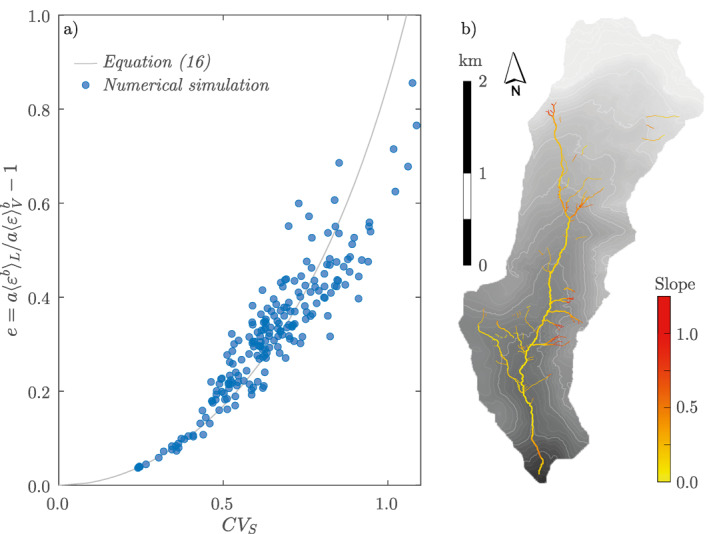
Estimation bias as a function of the coefficient of variation of the slope (a). Each point represents a different 100 m reach of the Valfredda stream network. Note that a maximum overlap among different reaches of 40% was allowed in this analysis. In this example, b was set to 1.18 as suggested by Ulseth et al. (2019) for high‐energy streams (ε is systematically higher than 0.02 m^2^ s^−3^). Spatial map of the slope S of the stream network in the Valfredda catchment (b).

Figure 3a shows e as a function of the coefficient of variation of the slope, $CV_{S}$, for the 200 sample reaches of the Valfredda creek. The simulated aggregation bias follows the general trend predicted by Equation 16, with a nonlinear increase of e with $CV_{S}$. In approximately 30% of the cases e>60%, with e peaking at 80% when $CV_{S}\approx 1$. The scatter of the numerical points around the theoretical equation is generated by deviations of the slope pdf from the gamma distribution. This happens more often in the most heterogeneous reaches, where the step and pool configuration generates a bimodal distribution of S.

## 4 Discussion

The main goal of this study is to clarify analogies and differences between local and reach‐scale k(ε) relations. To this aim, a simple hydrodynamic model was proposed in which the spatial variations of ε and k are linked to the heterogeneity of the slope. While the formulation is quite general, the suitability of the proposed model to describe the water flow in mountain reaches needs to be carefully assessed case‐by‐case. In these settings, in fact, the flow can be tortuous and irregular, thereby challenging the use of a simplified 1D framework. Several studies suggested the use of an updated version of the classical Gauckler‐Manning equation to model water flows in small mountain streams in which the Manning coefficient varies with the hydraulic radius (see Marcus et al., 1992). The proposed model could incorporate the effect of this dependence of the roughness on $R_{H}$ by properly tuning the coefficients ξ and γ in Equation 1. Furthermore, local energy dissipations with enhanced gas evasion could be observed in correspondence of hydraulic jumps (which are not described by our model), owing to the transition from supercritical and subcritical flow. While we acknowledge that 2D and 3D formulations might be more flexible in describing the spatial heterogeneity of complex flow fields typically observed in high‐energy streams (including those observed in presence of, for example, step and pools, hydraulic jumps, and abrupt planar discontinuities), we propose that the concept of aggregation bias introduced in this paper is quite general. In particular, our study indicates that—regardless of the specific features of the flow field—internal heterogeneity of the energy dissipation processes generates a potential bias in spatially integrated k(ε) laws. The magnitude of the bias is eventually driven by the local scaling exponent b and the underlying pdfs of the turbulent kinetic energy dissipation rate in the focus reach (Equations 10 and 14), which could be evaluated on a case‐by‐case basis. Therefore, the proposed framework not only provides a first‐order assessment of the impact of hydrodynamic heterogeneity on the outgassing of streams with variable slope, but offers a robust conceptual basis for evaluating the aggregation bias in many other settings, in which the major assumptions of the hydraulic model developed here are not fulfilled.

Our analytical results indicate that the aggregation of spatially heterogeneous flow conditions generates reach‐scale k that are higher (or slightly lower) than the transfer rates that would be observed in a homogeneous system with the same mean turbulent kinetic energy dissipation rate if b>0.5 (or b<0.25). The magnitude of the aggregation bias, seen as the relative difference in the mass transfer rate of the homogeneous and the heterogeneous system for a given value of the mean turbulent kinetic energy dissipation rate, mainly depends on two factors: (a) the scaling exponent of the local k versus ε law, with larger biases observed for b>0.5; and (b) the coefficient of variation of the local slope. In particular, for b>1 (which is the observed apparent scaling exponent for high‐energy streams) and $CV_{S}>1$ (as frequently observed in steep headwaters) the bias exceeds 100%. These results are also confirmed by the numerical simulations performed within 200 reaches belonging to a small headwater catchment of the Italian Alps. The numerical results demonstrate the robustness of our analytical results and the reliability of the assumption made about the distribution of the slope. Overall, our analysis reveals that theoretical local k(ε) relations cannot be straightforwardly inferred from aggregated k versus ε data points in high‐energy streams (where b>1) unless the system is internally homogeneous, which is a very rare circumstance in most real‐world settings.

The practical implications of the theoretical results shown in the paper are manyfold. First of all, the apparent scaling exponent that might be deduced from multiple reach scale tracer studies in high‐energy streams could be different from the underlying scaling exponent of the local k versus ε relation, especially whenever $CV_{S}$ is a function of ε, that is, if more energetic streams are characterized by a different degree of internal heterogeneity as compared to less energetic streams. Therefore, regional estimates and available k databases should be analyzed with caution as they could be affected by systematic aggregation biases. Second, reach‐scale k estimates pertaining to streams characterized by a different degree of heterogeneity might not be directly comparable: in high‐energy streams, where empirical data suggest values of b larger than 1, more heterogeneous streams will be featured by relatively larger gas transfer rates for a given value of the total kinetic energy dissipation rate. This might also be one of the reasons that underlie the observed scatter in the reach‐scale experimental k versus ε pairs in Ulseth et al. (2019). Understanding how reach‐wise scaling laws originate from local k(ε) relations is also important for spatial extrapolations of gas transfer velocities, which lie at the basis of regional or global‐scale studies on greenhouse gas evasion from streams and rivers. Our analysis indicates that spatial extrapolations of k should be performed taking into account the internal heterogeneity of streams: river reaches with the same mean energetic level could be in fact characterized by highly heterogeneous gas transfer velocities, if they are internally different. Thus, small‐scale morphological heterogeneity of low‐order streams (e.g., steps, pools, and cascades) arguably represents an important missing component of in‐stream nutrient cycling, which has relevant implications for the quantification of gas evasion from inland freshwater systems.

## 5 Conclusion

In this study, a simplified hydrodynamic model was used to analyze how a local power‐law relation between mass transfer velocity and energy dissipation rate can be altered when it is spatially integrated along a heterogeneous stream, and then referred to equivalent reach‐scale values of k and ε. Analytical derivations and numerical simulations indicate that local k(ε) power‐law relations are not preserved at the reach (or network) level in presence of significant internal spatial variations of the energy dissipation rate. In particular, a systematic positive aggregation bias is observed when the exponent of the local k versus ε relation, b is larger than 0.5, as typically observed in high‐energy streams. In these settings, the presence of spatially heterogeneous flow conditions generates reach‐scale values of k that are higher than the corresponding mass transfer rates that would be observed in a homogeneous system with the same mean turbulent kinetic energy dissipation rate. The magnitude of the bias can be as high as 100% if b>1 and the standard deviation of the slope exceeds the mean slope (i.e., $CV_{S}>1$). On the other hand, our results indicate that the aggregation bias is negative and negligible when the scaling exponent of the k versus ε relation is lower than 0.5, as predicted by surface renewal theories and empirically observed in low‐energy streams. Therefore, available k datasets should be analyzed with caution as the presence of aggregation biases in high‐energy streams might impair their use for the quantification of global‐scale gas evasion rates from rivers.

**Symbol List: Notation**
〈⋅〉
: Spatially averaged quantity.
A
: Cross‐sectional area ($L^{2}$).
a
: Proportionality coefficient of the local k(ε) relation ($T^{3b-1}/L^{2b-1}$).
b
: Power‐law scaling exponent of the local k(ε) relation (−).
C
: Gas concentration in water ($M/L^{3}$).
$C_{0}$
: Gas concentration at the reach inlet ($M/L^{3}$).
$C_{a}$
: Equilibrium gas concentration in water ($M/L^{3}$).
D
: Molecular diffusivity ($L^{2}/T$).
$D_{x}$
: Hydrodynamic dispersion ($L^{2}/T$).
d
: Hydraulic coefficient related to the uniform flow equation (−).
e
: Relative aggregation bias (−).
g
: Gravitational acceleration ($L/T^{2}$).
h
: Local water depth (stage) (L).
k
: Gas transfer velocity (L/T).
$k_{eq}$
: Reach‐wise equivalent gas transfer velocity (L/T).
L
: Reach length (L).
n
: Manning's coefficient ($T/L^{1/3}$).
P
: Total power dissipated along the reach ($M\cdot L^{2}/T^{3}$).
$p_{S}$
: pdf of the reach slope (−).
$p_{L}$
: Longitudinal pdf of the turbulent energy dissipation rate ($T^{3}/L^{2}$).
$p_{V}$
: Volumetric pdf of the turbulent energy dissipation rate ($T^{3}/L^{2}$).
Q
: Water discharge ($L^{3}/T$).
$R_{H}$
: Hydraulic radius (L).
S
: Local slope (−).
Sc
: Schmidt number (−).
t
: time (T).
U
: Local uniform flow velocity (L/T).
W
: Uniform reach width (L).
x
: longitudinal coordinate along the flow path (L).
α
: Scale parameter of the pdf of the slope (−).
β
: Shape parameter of the pdf of the slope (−).
ε
: Local turbulent kinetic energy dissipation rate ($L^{2}/T^{3}$).
$\varepsilon_{1}$
: Local turbulent kinetic energy dissipation rate for unit slope ($L^{2}/T^{3}$).
$\varepsilon_{eq}$
: Reach‐wise equivalent energy dissipation rate ($L^{2}/T^{3}$).
${\langle \varepsilon \rangle }_{L}$
: Longitudinal average of ε ($L^{2}/T^{3}$).
${\langle \varepsilon \rangle }_{V}$
: Volumetric average of ε ($L^{2}/T^{3}$).
Γ(⋅)
: Gamma function.
η
: Exponent of the hydraulic radius in the uniform flow equation (−).
ν
: Kinematic viscosity ($L^{2}/T$).
ξ
: Friction coefficient ($L^{1-\eta }/T$).
ρ
: Water density ($M/L^{3}$).
ω
: Wetted perimeter of the cross section (L).

## Supporting information

Supporting Information S1

## Data Availability

Slope data for the numerical simulations are available at Botter et al. (2021).
