# Supplementary material for: Heterogeneity Matters: Aggregation Bias of Gas Transfer Velocity Versus Energy Dissipation Rate Relations in Streams
Source: Geophys Res Lett. 2021 Sep 8;48(17):e2021GL094272. doi: 10.1029/2021GL094272 (PMC9286590; doi:10.1029/2021GL094272)
Supplement: Supplementary file 1 — Supporting Information S1 [file GRL-48-0-s001.pdf]

Supporting Information for "Heterogeneity matters: aggregation bias of reach-wise gas transfer velocity vs. energy dissipation rate relationships"

Gianluca Botter[1], Paolo Peruzzo[1], Nicola Durighetto[1]

[1]Department of Civil, Environmental and Architectural Engineering, University of Padua, Italy.

S1. Formulation of $p_V(\varepsilon)$

The explicit formulation of $p_V(\varepsilon)$ reported in equation (7) of the main text can be easily obtained once $h(\varepsilon)$ and $\langle h \rangle$ are known, by taking advantage of the relation

$$p_V(\varepsilon) = p_L(\varepsilon)\frac{h(\varepsilon)}{\langle h \rangle}\,. \tag{1}$$

First, we explicit $h(\varepsilon)$ by combining equations (2) and (4) of the main text:

$$h(\varepsilon) = \left(\frac{Q}{\xi W}\right)^{2d}\left(\frac{\varepsilon}{\varepsilon_1}\right)^{-\frac{d}{1+d}}\,. \tag{2}$$

Then, $\langle h \rangle$ is calculated. Starting from equations (2) and (5) of the main text, which respectively express $h(S)$ and $p_S(S)$, with a derived distribution approach the pdf of water depth h can be formulated as

---

Corresponding author: G. Botter (gianluca.botter@dicea.unipd.it)

$$p_h(h) = \frac{\alpha^{-\beta}}{d}\left(\frac{Q}{\xi W}\right)^{2\beta}\frac{1}{\Gamma(\beta)}h^{-\frac{\beta}{d}-1}exp\left(-\frac{1}{\alpha}\left(\frac{Q}{\xi W}\right)^{2}h^{-\frac{1}{d}}\right). \quad (3)$$

Note that this is not a generalized gamma distribution due to the negative exponent of the random variable h. The mean water depth can be calculated from equation (3) as:

$$\langle h\rangle = \int_0^\infty hp_h(h)dh = \frac{\alpha^{-d}}{\Gamma(\beta)}\left(\frac{Q}{\xi W}\right)^{2d}\Gamma(\beta-d). \quad (4)$$

Finally, combining equations (1), (2) and (4) we obtain $p_V(\varepsilon)$ as reported in equation (7) of the main text.

S2. Estimation bias e

The estimation bias e is calculated starting from the bth order moment of ε, $\langle\varepsilon^b\rangle_L$, and the bth power of the volumetric mean of ε, $\langle\varepsilon\rangle_V{}^b$. Given that $p_L(\varepsilon)$ takes the form of a generalized gamma distribution, as shown in the main text by equation (6), the bth order moment of ε is

$$\langle\varepsilon^b\rangle = \varepsilon_1^b\alpha^{b(1+d)}\frac{\Gamma(\beta+b(1+d))}{\Gamma(\beta)}. \quad (5)$$

Similarly, $\langle\varepsilon\rangle_V$ can be derived from $p_V(\varepsilon)$, which is also a generalized gamma distribution:

$$\langle\varepsilon\rangle_V = \varepsilon_1\alpha^{1+d}\frac{\Gamma(\beta+1)}{\Gamma(\beta-d)}. \quad (6)$$

Equations (5) and (6) can be easily combined to obtain the formulation of the estimation bias shown in equation (16) of the main text.
